# Fe_3_O_4_-graphene oxide nanocomposites functionalized with hyaluronic acid and folic acid as dual pH/NIR-responsive platforms for synergistic chemophotothermal therapy of breast cancer

**DOI:** 10.1039/d5ra08760k

**Published:** 2026-03-02

**Authors:** Bin Jia, Yimu Zhong, Danyang Zhai, Jing Pang, Bo Sha, Na Li, Bo Li, Wenting Liang, Wei Bian

**Affiliations:** a Department of Chemistry, School of Basic Medical Science, Shanxi Medical University Taiyuan 030001 China bjia2006@163.com; b Academy of Medical Sciences, Shanxi Medical University Taiyuan 030001 China; c Institute of Environmental Science, Department of Chemistry, Shanxi University Taiyuan 030006 China

## Abstract

Chemotherapy consistently exhibits constrained therapeutic efficacy due to inadequate tumor specificity and unavoidable multidrug resistance. However, the development of nanoscale drug carrier systems offers a promising therapeutic approach for cancer treatment, given their proven potential in targeted delivery and synergistic therapeutic outcomes. In this work, nanoparticles (NPs) designed for drug delivery and photothermal therapy (PTT) were fabricated by grafting hyaluronic acid (HA) and folic acid (FA) onto the surface of Fe_3_O_4_-modified graphene oxide (MGO). These MGO-HA-FA NPs demonstrated specific dual-targeting functionality for both CD44 and folate receptors. Meanwhile, the antitumor drug doxorubicin hydrochloride (DOX) was efficiently encapsulated by MGO-HA-FA and the drug loading capacity (DLC) could reach 58 wt%. And MGO-HA-FA demonstrated favorable biocompatibility, low systemic toxicity, and high photothermal conversion efficiency. Furthermore, upon near-infrared radiation (NIR) laser irradiation, DOX/MGO-HA-FA nanoparticles could *in situ* ablate tumor cells and further trigger drug release. This process demonstrated a remarkable synergistic therapeutic efficacy combining photothermal therapy and chemotherapy, achieving a tumor inhibition rate > 90%. When synergistically combined with photothermal therapy, this nanocomposite exhibits significantly enhanced antitumor efficacy against 4T1 tumor-bearing murine models relative to monotherapeutic approaches. This improvement is attributable primarily to its precisely controlled, tumor-targeted drug release mechanism and significantly reduced systemic toxicity. Thus, this study proposed a novel active dual-targeted chemophotothermal therapy (CPT) nanocarrier for targeted cancer therapy.

## Abstract 2

Schematic illustration of the synthesis and therapeutic mechanism of DOX/MGO-HA-FA.

## Graphical abstract

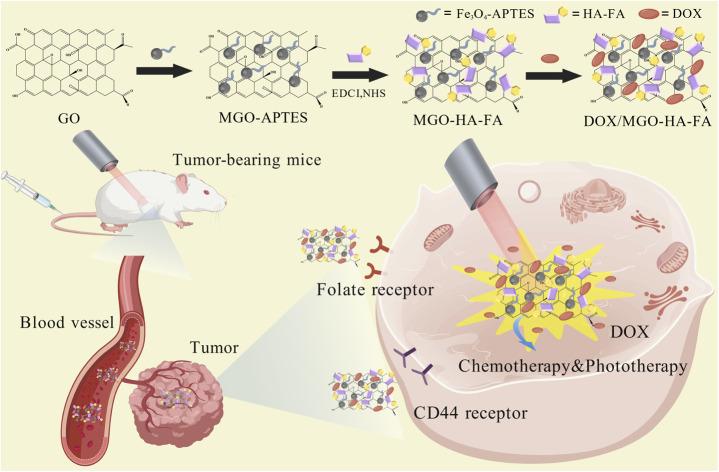

## 1. Introduction

Cancer ranks as the leading cause of death in most countries of the world.^1,2^ Currently, the global burden of disease attributed to cancer incidence and mortality continues to increase.^3^ In the clinical management of cancer, the primary conventional therapeutic modalities encompass surgical intervention, radiation therapy, chemotherapy, and immunotherapy.^4–6^ Among these treatments, chemotherapy is the main, current method to kill tumor cells.^7,8^ Doxorubicin (DOX) is one of the most frequently utilized chemotherapeutic drugs, with broad application in treating diverse malignancies including bladder cancer, breast cancer, cervical cancer, gastric cancer, leukemia, and lung cancer.^9,10^ Unfortunately, the clinical use of DOX can induce cardiac toxicity, liver damage, and bone marrow suppression, stemming from its low selectivity for tumor tissues.^11–13^ Studies have demonstrated that nanomaterial-based drug delivery systems offer the advantages of targeted therapy and reduced drug side effects.^14–18^ And research conducted by Ximing Yang *et al.* and Lin Qin *et al.* indicated that the emergence of a combinatorial therapy mode effectively inhibits primary and metastatic tumors and reduces tumor recurrence.^19,20^ Thus, there is an urgent need to develop a drug delivery system to transport DOX to the tumor site and improve therapeutic efficacy through synergistic therapy. Chemophotothermal therapy (CPT), the integration of chemotherapy and photothermal therapy (PTT), is of particular interest as it can facilitate the thermal ablation of tumor cells and trigger long-term chemical inhibition—with PTT achieving thermal ablation by converting near-infrared (NIR) laser energy into heat (>42 °C).^21,22^ At the same time, Zhuang Liu and Guangyu Zhu's research groups have shown that near-infrared light has high tissue penetration depth and minimal damage to normal tissue.^23,24^ Thus, developing an effective CPT nanoplatform is highly desirable, and the photothermal agents are the key point. Various nanomaterials, such as copper-based, gold, manganese dioxide, graphene oxide, and magnetite nanomaterials, have garnered increasing attention in photothermal therapy (CPT) due to their remarkable near-infrared (NIR) absorption characteristics.^25–27^ Among these, graphene oxide (GO) exhibits significant promise for applications in drug delivery and PTT therapeutic modalities, owing to its sizeable specific surface area and superior near-infrared (NIR) light absorptive properties.^28–33^ Notably, Fe_3_O_4_ is recognized for its low biotoxicity, distinct superparamagnetization, and effective photothermal performance in photo-mediated cancer therapy.^34,35^ Based on previous studies by our research group, combining Fe_3_O_4_ with GO can efficiently load small-molecule drugs and offer more conspicuous NIR absorption characteristics, and it is a suitable nanocarrier for photothermal therapy.^36,37^ Although they can actively accumulate in tumor regions through the effect of enhancing permeability and retention (EPR) effect, insufficient intracellular uptake remains a major challenge.^38–40^ To overcome this limitation, the development of efficient drug delivery systems with enhanced targeting efficiency is of great significance for achieving better therapeutic performance. As shown by Mariana Scaranti, Qi Xiong, Bethany M. Cooper *et al.*, endocytosis mediated by ligands specific to cancer cells confers active targeting properties on drug delivery systems, which in turn enhances the accuracy and effectiveness of carcinoma treatment.^41–43^ For instance, the *trans*-membrane glycoprotein CD44, whose expression is markedly elevated in malignant cells, can be selectively recognized and bound by hyaluronic acid (HA), an endogenous anionic polysaccharide of high molecular weight.^42,44^ Folate receptors (FR), frequently amplified on neoplastic plasma membranes, are selectively engaged by folic acid (FA), a water-soluble vitamin indispensable for nucleotide biosynthesis in rapidly dividing cells, thereby enabling FR-mediated tumor targeting.^45,46^ More importantly, CD44 receptors and FR have been reported to be co-expressed in several tumor cells.^47,48^ The integration of various types of targeting ligands can boost cellular internalization, which in turn enhances the ability of drugs to enter cells to some extent.^49^ As reported in a recent study by Ru Zhang *et al.*, functionalizing drug delivery systems with HA-linked FA polymers enables satisfactory targeting through receptor-ligand-mediated endocytosis, significantly boosting drug internalization by specific tumor cells.^50^

Hence, a multitargeted nano-drug delivery system termed MGO-HA-FA was engineered herein by functionalizing magnetic graphene oxide (MGO) with hyaluronic acid (HA) and folic acid (FA) ligands. It possesses steady photothermal properties, great biosafety, and high loading capacities. Upon near-infrared radiation (NIR) laser irradiation, DOX/MGO-HA-FA nanoparticles exhibited maximal inhibitory effects on cancer cells. By integrating chemotherapy with photothermal therapy, DOX/MGO-HA-FA induced nearly complete eradication of subcutaneous tumors in mice within a 14-day treatment period. Biocompatibility assessments of the nanoplatform demonstrated this therapeutic strategy to be a safe and well-tolerated treatment regimen, with no observable systemic toxicities. Our results demonstrate significant potential for this approach to serve as a combined chemotherapy and photothermal therapy modality in future cancer treatment.

## 2. Materials and methods

### 2.1. Preparation of MGO-HA-FA and DOX/MGO-HA-FA

The dual-targeted nanocomposite MGO-HA-FA was synthesized by covalently conjugating HA-FA polymer to aminated magnetic graphene oxide (MGO-APTES) through 1-ethyl-3-(3-dimethylaminopropyl)carbodiimide-*N*-hydroxysuccinimide (EDCI-NHS)-mediated carboxyl activation, followed by purification *via* magnetic separation and freeze-drying. For the preparation of DOX-loaded nanoparticles (DOX/MGO-HA-FA), MGO-HA-FA was incubated with DOX solution in PBS (pH 7.4) under dark conditions, with unbound drug removed by magnetic separation and washing steps. Detailed synthetic procedures are available in SI File S2.

### 2.2. Characterization techniques

Transmission electron microscopy (TEM, FEI Tecnai G2 F30, Japan) and scanning electron microscopy (SEM, ZEISS Sigma 300, Germany) provided morphological insights into MGO and MGO-HA-FA nanoparticles. X-ray diffraction (XRD, Rigaku SmartLab SE, Japan) was used to characterize the crystalline structure of the composites, while Raman spectra (Horiba LabRAM HR Evolution, Japan) were acquired to analyze the carbon skeleton structure and intermolecular interactions. For particle size, zeta potential, and polydispersity index (PDI)—with PDI assessing dispersion uniformity in solution—a Zetasizer Nano ZSE (Malvern, UK) was employed. Fluorescence spectral analysis was carried out using an F-7100 spectrophotometer (Hitachi, Japan). Fourier transform infrared (FTIR) spectra were recorded over the range of 500–4000 cm^−1^ with a Bruker INVENIO spectrometer (Germany). Thermogravimetric analysis (TGA) was performed on a TA Instruments Q50 system (coupled with Hitachi 7200, Japan) under a nitrogen flow, with a heating rate of 10°C min^−1^ from ambient temperature to 800 °C. UV-vis absorption spectra of 0.25 mg per mL GO, MGO, and MGO-HA-FA dispersions were acquired using a UH5300 spectrophotometer (Japan). For the Cell Counting Kit-8 (CCK-8) assay, absorbance values were measured with a SpectraMax190 microplate reader (USA). We conducted fluorescence imaging with an Olympus FV3000 laser confocal microscope (Japan), while infrared thermal images were recorded using an FLIR PTi120 thermal camera.

### 2.3. *In vitro* photothermal performance of MGO-HA-FA

Under 808 nm laser irradiation, the photothermal behavior of MGO-HA-FA was characterized. Briefly, 1 mL aliquots of MGO-HA-FA solutions at concentrations of 0.1, 0.4, 0.8, and 1.0 mg mL^−1^ (with PBS as a control) underwent 5-minute exposure to an 808 nm NIR laser (2.0 W cm^−2^), with thermal imaging used to record temperature shifts every minute. Additionally, a 1 mL sample of MGO-HA-FA solution (1.0 mg mL^−1^) was exposed to 808 nm laser at power densities of 1.0, 1.5, and 2.0 W cm^−2^ for 5 min to monitor real-time temperature variations. Photothermal stability was assessed by recording temperature fluctuations of the 1 mL 1.0 mg mL^−1^ dispersion over 3 cycles of 20-minute 808 nm laser (2 W cm^−2^) on/off irradiation.

Additionally, the photothermal conversion performance of MGO-HA-FA nanoparticles was evaluated using the classic laser-induced heating-cooling method. The photothermal conversion efficiency (*η*) was calculated *via* the classic laser-induced heating-cooling method, with the values determined using formula (S1).

### 2.4. Drug loading experiment

#### 2.4.1. Drug loading kinetics study

A fluorescence-based standard curve for DOX was established using an excitation wavelength of 490 nm and measuring emission at 558 nm. For drug loading kinetic analysis, 1.0 mg MGO-HA-FA nanoparticles were dispersed *via* ultrasonication into 4 mL of 0.01 mg per mL DOX solution, which was followed by shaking at 200 rpm under dark conditions at 37 °C. At chosen time intervals, magnetic separation was used to collect supernatants, whose fluorescence intensity at 558 nm was measured; these data were then used to derive DOX loading capacity. Adsorption kinetics were analyzed using Lagergren's pseudo-first-order and Ho's pseudo-second-order models (eqn (S1) and (S2)).

#### 2.4.2. Isothermal adsorption experiment

For the determination of drug loading capacity, 1.0 mg of MGO-HA-FA was incubated with 4 mL of DOX solutions (0.01–0.5 mg mL^−1^) and shaken at 200 rpm in darkness for 24 hours. After centrifugation, the fluorescence emission of the appropriately diluted supernatant (*λ*_ex_ = 558 nm) was recorded, and the drug loading capacity (DLC) was then back-calculated from the obtained intensity according to formula (S2). The adsorption isotherm was analyzed using the Langmuir (eqn (S3)) and Freundlich (eqn (S4)) models to evaluate the binding mechanism.

### 2.5. DOX release behaviors of DOX/MGO-HA-FA NPs

#### 2.5.1. pH-triggered drug release

The pH-responsive drug release behavior of DOX/MGO-HA-FA was evaluated at 37 °C in PBS buffers (pH 5.3, mimicking intracellular tumor microenvironment; pH 7.4, simulating physiological conditions). Briefly, 4.0 mL PBS (at each pH level) was used to suspend 1.0 mg DOX/MGO-HA-FA, which was then shaken continuously at 200 rpm in the dark. At predetermined time intervals, supernatants were collected *via* magnetic separation, and DOX concentration was quantified by fluorescence measurement to determine release profiles.

#### 2.5.2. NIR-triggered drug release

The NIR-responsive release of DOX from DOX/MGO-HA-FA was assessed *in vitro* using PBS (pH 5.3) at 37 °C, with and without NIR irradiation (808 nm, 2 W cm^−2^). Regarding the irradiated cohort, 5 min of NIR exposure was applied prior to supernatant separation; the non-irradiated group served as control. DOX concentration in supernatants was quantified to calculate drug release, with release rates determined using formula (S3).

### 2.6. Hemolysis test

*In vitro* hemolysis tests were performed to assess the compatibility of MGO-HA-FA and DOX/MGO-HA-FA with red blood cells (RBCs). After centrifugation of rat blood at 3000 rpm for 30 minutes to isolate RBCs, the cells were washed with PBS until the supernatant was clear and resuspended in PBS. Aliquots (200 µL) of the RBC suspension were incubated with 1.0 mL of test solutions—deionized water (positive control), PBS (negative control), and MGO-HA-FA/DOX/MGO-HA-FA at concentrations of 0.01, 0.02, and 0.03 mg mL^−1^—for 2 h at 37 °C. After centrifugation (3000 rpm, 10 min), supernatant absorbance was measured at 541 nm (hemoglobin absorption maximum). The percentage of hemolysis was determined by the equation: [(sample absorbance – negative control absorbance)/(positive control absorbance – negative control absorbance)] × 100.

### 2.7. Anti-tumor activity of MGO-HA-FA *in vitro*

MCF-7 (CD44^+^/folate receptor^+^), MDA-MB-231 (CD44^+^/folate receptor^+^), and A549 (CD44^+^/folate receptor^−^) cells were used for *in vitro* studies. They were maintained in RPMI 1640, DMEM-high glucose, and McCoy's 5A media, respectively, each supplemented with 10% fetal bovine serum (FBS) and 1% penicillin–streptomycin. Time-dependent and receptor-blocked intracellular uptake of DOX/MGO-HA-FA was visualized *via* confocal microscopy. Cytotoxicity of MGO-HA-FA and DOX/MGO-HA-FA (with/without 808 nm NIR irradiation, 2 W cm^−2^) was assessed using CCK-8 assays, and live/dead cell discrimination was performed *via* Calcein-AM/PI staining. Detailed protocols and group settings are available in SI File S5.

### 2.8. *In vivo* antitumor therapeutic efficacy

#### 2.8.1. Animal studies

All animal procedures were performed in accordance with the Guidelines for the Care and Use of Laboratory Animals of Shenzhen University and approved by the Institutional Animal Care and Use Committee (IACUC).

Six-week-old female BALB/c mice were purchased from SPF (Beijing) Biotechnology Co., Ltd. All mice were housed in specific pathogen-free (SPF) barrier facilities at the Laboratory Animal Center of Shanxi Medical University. The mice were maintained under a 12 h light/12 h dark cycle at 22–26 °C, with free access to sterile pelleted food and water. All animal protocols used in this study were approved by the IACUC of Shanxi Medical University.

4T1 cells were collected and washed twice with cold phosphate-buffered saline (PBS). A suspension of 3 × 10^6^ 4T1 cells in cold PBS was injected into the axillary region of the mice. Every two days, the length and width of the tumors were measured using vernier calipers, and the tumor volume was calculated using the following formula: tumor volume (mm^3^) = 1/2 × length (*L*) × width (*W*). The body weight of the animals was measured with an electronic balance every two days. Mice with a tumor volume of approximately 100 mm^3^ were selected for the formal animal experiment.

#### 2.8.2. Antitumor efficacy assessment

In the biosafety evaluation cohort, 21 tumor-bearing mice (*n* = 3 per group) had their body weight and tumor volumes serially assessed at two-day intervals between day 0 and day 14 post-ablation. Upon euthanasia, tumors were removed, weighed, and the collected data stored for analysis.

#### 2.8.3. Biosafety evaluation

To further confirm the safety of the therapeutic approach, 21 tumor-bearing mice (*n* = 3 per group) had their body weights recorded every other day from day 0 to 14 post-irradiation as an indicator of metabolic status. On day 14, mice were euthanized; major organs were excised for hematoxylin and eosin (H&E) staining, and blood samples acquired *via* eyeball enucleation were assessed for complete blood cell counts (WBC, white blood cell count; RBC, red blood cell count; PLT, platelet count; HGB, hemoglobin) and serum biochemical markers (ALT, alanine transaminase; ALB, albumin; AST, aspartate transaminase; UREA, urea; CREA, creatinine; TP, total protein) to determine the biosafety of DOX/MGO-HA-FA.

### 2.9. Data analysis

Quantitative data were analyzed using SPSS 25.0, with results reported as mean ± standard deviation (SD). Group differences were determined *via* one-way analysis of variance (ANOVA), and statistical significance was established at *P < 0.05, **P < 0.01, and ***P < 0.001.

## 3. Results and discussion

### 3.1. Structural characterization of MGO-HA-FA

**Fig. 1:**
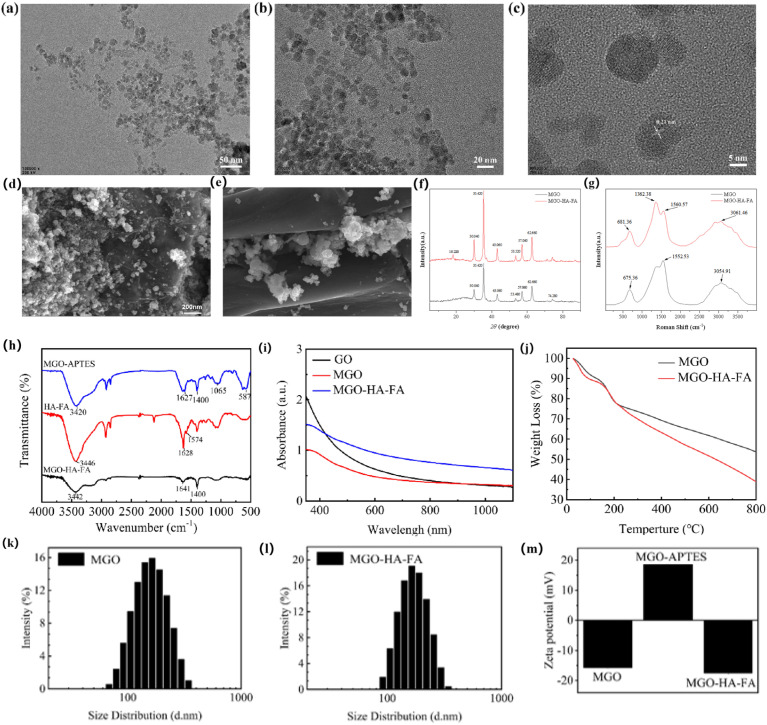
TEM images of MGO (a) and MGO-HA-FA (b); the high-resolution TEM (HRTEM) image of MGO-HA-FA (c); SEM images of MGO (d) and MGO-HA-FA (e); XRD spectram of MGO and MGO-HA-FA (f); Roman spectram of MGO and MGO-HA-FA (g); FTIR spectram of MGO-APTES, HA-FA and MGO-HA-FA (h); UV-vis absorption spectrums of 0.25 mg per mL GO, MGO and MGO-HA-FA solutions (i); the thermogravimetric (TG) curves of MGO and MGO-HA-FA (j); size distribution of the aqueous solution of MGO (k) and MGO-HA-FA (l); zeta potential of MGO, MGO-APTES and MGO-HA-FA (m).

Transmission electron microscopy (TEM) was employed to evaluate the topographical architecture and dimensions of both pristine MGO and the dual-targeted MGO-HA-FA nanoconstruct, with representative micrographs provided in Fig. 1a, GO is a layered structure located at the bottom layer; Fe_3_O_4_ nanoparticles exhibited a spherical morphology with a diameter of around 12 nm and coated on its surface. In addition, Fig. 1b presents the image of MGO-HA-FA, the conjugation of HA and FA had no impact on the shape or dimensions of MGO. Ultrastructural analysis (Fig. 1c) by high-resolution TEM (HRTEM) evidences crystalline Fe_3_O_4_ domains within MGO-HA-FA nanoparticles, displaying a well-resolved lattice fringe of 0.21 nm that is consistent with the (400) plane of magnetite.

The SEM images of MGO and MGO-HA-FA are presented in Fig. 1d and e, respectively. In Fig. 1d, the typical wrinkled lamellar structure of GO is distinctly visualized, with Fe_3_O_4_ nanoparticles uniformly distributed across the GO sheets. These Fe_3_O_4_ nanoparticles exhibit a quasi-spherical nanostructure; however, partial aggregation of Fe_3_O_4_ particles is observed, which can be ascribed to the magnetic interactions between adjacent particles. Following modification with HA-FA (Fig. 1e), the lamellar architecture of GO remains well-preserved, and the Fe_3_O_4_ particles are also retained in the composite. Notably, the dispersibility of Fe_3_O_4_ particles is further enhanced relative to that in the pristine MGO.

The XRD patterns of MGO and MGO-HA-FA are depicted in Fig. 1f. The diffraction peaks of MGO can be perfectly indexed to the standard card of Fe_3_O_4_ with a cubic spinel structure (JCPDS no. 19-0629). Specifically, the diffraction peaks at 2*θ* ≈ 30.1°, 35.5°, 43.1°, 53.4°, 57.0° and 62.6° are assigned to the (220), (311), (400), (422), (511) and (440) crystal planes of Fe_3_O_4_, respectively. The presence of these characteristic peaks confirms that crystalline Fe_3_O_4_ nanoparticles have been successfully grown *in situ* on the surface of the GO matrix. Notably, the characteristic diffraction peak of pristine GO corresponding to the (001) crystal plane at 2*θ* ≈ 9.0° is not prominent in the XRD pattern of MGO. This is a common phenomenon in Fe_3_O_4_/GO composites, which can be ascribed to the disruption of the ordered stacking structure of GO sheets by the densely loaded Fe_3_O_4_ nanoparticles. All the characteristic diffraction peaks of Fe_3_O_4_ are retained in the XRD pattern of MGO-HA-FA, which are consistent with those of MGO, indicating that the crystalline structure of Fe_3_O_4_ remains intact during the HA-FA modification process. No new sharp diffraction peaks appear in the XRD pattern of MGO-HA-FA, which is because both HA and FA are amorphous components, and amorphous organic substances generally do not produce distinct crystalline diffraction peaks in XRD spectra. In addition, the intensity of Fe_3_O_4_ diffraction peaks in MGO-HA-FA is slightly lower than that in MGO. This is due to the coverage of the amorphous HA-FA layer on the surface of the composite, which weakens the X-ray diffraction signal of Fe_3_O_4_ nanoparticles. This observation further verifies the successful grafting of HA-FA onto the MGO surface.

The Raman spectra of MGO and MGO-HA-FA are presented in Fig. 1g. In the spectrum of MGO, the peak at 681.36 cm^−1^ corresponds to the Fe–O bond stretching vibration of cubic spinel-type Fe_3_O_4_. The presence of this peak directly confirms that Fe_3_O_4_ nanoparticles have been successfully loaded onto the GO matrix; the stable peak position and intensity further indicate that the crystalline structure of Fe_3_O_4_ remains intact during the GO composite formation process. The peaks at 1362.38 cm^−1^ (D band) and 1560.57 cm^−1^ (G band) are typical characteristic peaks of graphene oxide (GO): the D band corresponds to the defective/disordered structure of sp^3^-hybridized carbon, while the G band is assigned to the in-plane graphitization vibration of sp^2^-hybridized carbon. The D/G intensity ratio of MGO is calculated to be approximately 1.23, which is higher than that of pristine GO (typically < 1.0). This result suggests that the coordination interaction between Fe_3_O_4_ and oxygen-containing functional groups on the GO surface introduces additional structural defects into the GO carbon skeleton. After modification with HA-FA, the peak at 675.36 cm^−1^ in the spectrum corresponds to Fe–O bond vibration; only a slight peak position shift (relative to the 681.36 cm^−1^ peak of MGO) is observed, demonstrating that the HA-FA modification process did not disrupt the crystalline structure of Fe_3_O_4_, thus preserving the magnetic responsiveness of the material. In the MGO-HA-FA spectrum, only the G band (at 1552.53 cm^−1^, corresponding to sp^2^-hybridized carbon) is observed, with no distinct D band (sp^3^ defective carbon); additionally, the intensity of this G band (9028.62) is significantly higher than that of the G band in MGO (5910.38). This phenomenon arises because the HA-FA polymer binds to the sp^3^ defective sites on the GO surface *via* hydrogen bonding and electrostatic interactions, which passivates some of the disordered structures while enhancing the integrity of the sp^2^ domain. The slight blue shift of the G band (from 1560.57 cm^−1^ to 1552.53 cm^−1^) further confirms the interaction between HA-FA and GO, verifying the successful realization of functional modification.

FT-IR spectra for MGO, HA-FA, and MGO-HA-FA are presented in Fig. 1h. For MGO's FT-IR spectrum, a broad, intense peak around 3426 cm^−1^ corresponds to O–H stretching vibrations, while peaks at 2924 and 2850 cm^−1^ are attributed to C–H stretching vibrations. The peak at 1630 cm^−1^ arises from C=C stretching vibrations of aromatic moieties, and the band at 1400 cm^−1^ is assigned to epoxy group stretching vibrations. Additionally, the peak at 573 cm^−1^ is associated with shifted Fe–O bond stretching vibrations,^51,52^ this confirms the successful modification of Fe_3_O_4_ on the GO surface. In the FT-IR spectrum of HA-FA, peaks at 3446 and 1628 cm^−1^ correspond to O–H (carboxyl groups) and N–H (amino groups), respectively, while the signal at 1574 cm^−1^ arises from C=O stretching vibrations—findings that validate HA-FA synthesis. Following further functionalization of MGO with HA-FA, peaks at 1603 cm^−1^ and 1396 cm^−1^ are assigned to the C=N and C–N bonds in HA-FA, respectively. The notable intensification of peaks between 1603–1153 cm^−1^ is attributed to HA-FA modification. In Fig. S2, graphene oxide shows a broad band at ∼3400 cm^−1^ (O–H stretch), along with peaks at ∼1720 cm^−1^ (C=O, carboxyl), ∼1620 cm^−1^ (sp^2^ C=C) and ∼1050 cm^−1^ (C–O, epoxy/hydroxyl), with these oxygenated groups providing reactive sites for covalent/noncovalent modification. Fe_3_O_4_ exhibits a distinct peak at ∼580 cm^−1^ (Fe–O stretch, spinel structure), confirming phase purity and magnetic responsiveness for targeted delivery. Folic acid displays characteristic peaks at ∼1700 cm^−1^ (C=O, carboxyl) and ∼1600/1500 cm^−1^ (aromatic C=C), which serve as structural fingerprints for folate-receptor-mediated tumor targeting. Hyaluronic acid shows bands at ∼3400 cm^−1^ (O–H), ∼1640 cm^−1^ (C=O, carboxyl) and ∼1050 cm^−1^ (C–O–C, glycosidic bond), verifying its polysaccharide architecture to support CD44-targeting and biocompatibility. Collectively, these results confirm the successful synthesis of MGO-HA-FA.

UV absorption spectra for GO, MGO, and MGO-HA-FA are presented in Fig. 1i. Notably, MGO-HA-FA showed significantly higher NIR absorbance compared to GO and MGO. This enhancement can be attributed to two factors: Fe_3_O_4_ itself exhibits some optical absorption in the NIR region, and HA-FA modification improves the dispersibility of MGO-HA-FA.

TGA results are shown in Fig. 1j, the weight loss observed in MGO may stem from the decomposition of graphene oxide and Fe_3_O_4_. Graphene oxide, in particular, contains abundant oxygenated functional groups that undergo cleavage during thermal treatment.^49^ Under identical conditions, MGO-HA-FA exhibited more pronounced weight loss. This disparity in weight loss between MGO-HA-FA and MGO arises from the decomposition of HA-FA, with the HA-FA modification amount calculated to be approximately 152 mg g^−1^.

The average hydrated particle size and zeta potential of MGO, dispersed in pure water at pH 7.4, were 163.3 nm (PDI = 0.258) (Fig. 1g) and −15.67 mV (Fig. 1k), respectively. The particle size of MGO-HA-FA, also dispersed in pure water at pH 7.4, was 189.1 nm (PDI = 0.293) (Fig. 1l). The increase in particle size in comparison with MGO was attributed to the successful loading of HA-FA conjugation on the surface of MGO. Furthermore, the zeta potential of MGO-HA-FA was −17.5 mV (Fig. 1m), which could maintain the stability of the carrier in blood circulation.

### 3.2. Photothermal effect of MGO-HA-FA

**Fig. 2:**
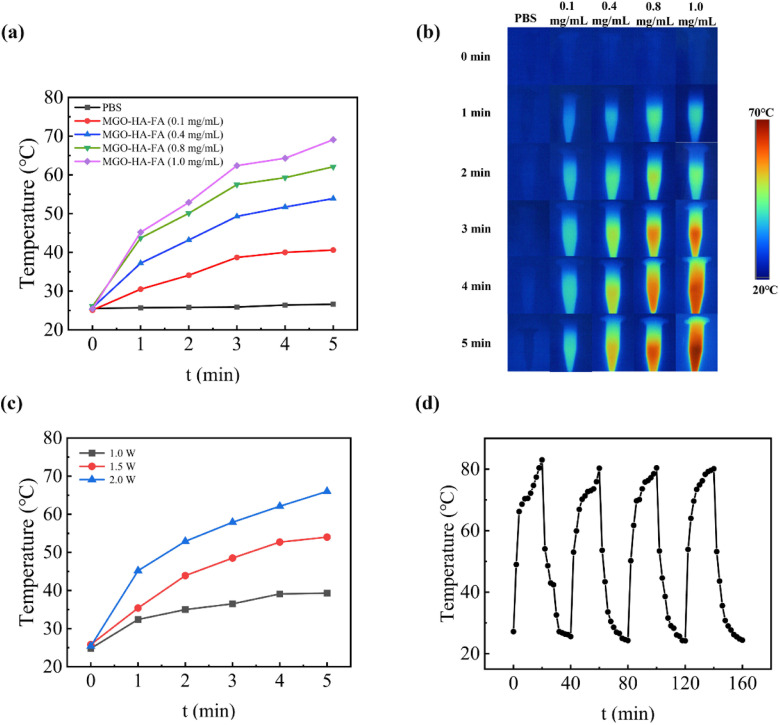
Temperature curves of different concentrations of MGO-HA-FA solutions under 808 nm laser irradiation (2 W cm^−2^) (a); infrared thermal images (b) of MGO-HA-FA solutions with different concentration obtained by using the thermal imaging system; temperature change curves of 1.0 mg per mL MGO-HA-FA under different laser intensities (808 nm) (c); temperature curves of MGO-HA-FA with three irradiation on/off cycles under 808 nm laser irradiation (d).

Fig. 2a reveals that MGO-HA-FA solutions exhibited a temperature rise with extended laser irradiation. When MGO-HA-FA concentrations increased from 0 to 1.0 mg mL^−1^, the maximum solution temperature rose from 26.4 °C to 69.1 °C after 5-minute exposure to an 808 nm NIR laser (2 W cm^−2^). In contrast, PBS maintained a stable room temperature with no significant fluctuations. Infrared thermal images (Fig. 2b) depict temperature variations over this 5-minute period.

Additionally, increasing the NIR laser intensity from 1.0 to 2.0 W cm^−2^ caused the temperature of 1.0 mg mL^−1^ MGO-HA-FA solutions to increase from 39.3 °C to 66 °C (Fig. 2c). Furthermore, Fig. 2d shows temperature rise/fall curves under 808 nm irradiation (on) and in its absence (off): temperature increased rapidly upon initial laser exposure, then gradually cooled to room temperature once irradiation stopped. Notably, photothermal performance showed no obvious degradation even after three laser on/off cycles, indicating good photothermal stability of MGO-HA-FA.

Moreover, the photothermal conversion efficiency of MGO-HA-FA was calculated to be 54.3% using the standard laser-induced heating-cooling method, further verifying its excellent photothermal conversion capability.

These results confirm that MGO-HA-FA nanoparticles can efficiently absorb light to generate heat, supporting their potential as effective photothermal agents for cancer cell elimination in photothermal therapy.

### 3.3. Drug loading behavior

**Fig. 3:**
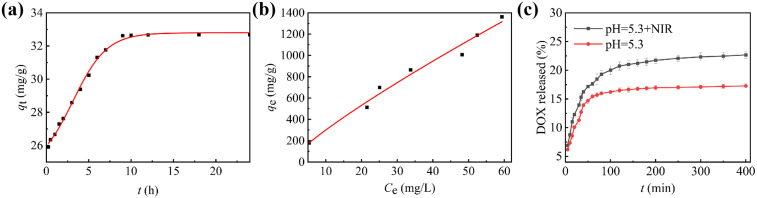
The pharmacokinetic curve of DOX on MGO-HA-FA (a); the adsorption isotherm curve for DOX loaded by MGO-HA-FA (b); the release amount of DOX on MGO-HA-FA in PBS buffer at pH 5.3 with or without laser irradiation (808 nm, 2 W cm^−2^) (c).

The standard concentration curve for DOX hydrochloride is presented in Fig. S3a, showing good linearity over the range of 5 × 10^−4^–5 × 10^−3^ mg mL^−1^ (*R*^2^ = 0.99131). The adsorption process reached a plateau after 9 hours (Fig. 3a). To investigate the adsorption mechanism, Lagergren's pseudo-first-order and Ho's pseudo-second-order kinetic models were applied. Linear fitting curves for these models and their respective kinetic parameters are provided in Fig. S3b, c and Table S1. The drug adsorption process was determined to be better described by Ho's pseudo-second-order model (*R*^2^ = 0.9996), indicating that chemical adsorption—such as hydrogen bonding and π–π interactions—serves as the primary driving force for the interaction between DOX and MGO-HA-FA.

At a DOX concentration of 0.4 mg mL^−1^, the loading amount of MGO-HA-FA reached 1326 mg g^−1^, with a drug loading capacity (DLC) of 58 wt%—a value significantly higher than those reported in previous studies.^53–55^ Additionally, comparison of the monolayer Langmuir model (Fig. S3d) and multilayer Freundlich adsorption model (Fig. S3e) revealed that the latter provided a better fit for the isothermal adsorption process (*R*^2^ = 0.92712), with parameters listed in Table S2. These findings indicate that DOX loading onto MGO-HA-FA is well-described by the multilayer Freundlich isotherm model.

### 3.4. DOX release of DOX/MGO-HA-FA

DOX release from DOX/MGO-HA-FA was profiled at 37 °C in PBS (pH 7.4 *vs.* 5.3) to mirror extracellular and tumor endo-lysosomal conditions. The percentage of drug released at various time points under these pH conditions is presented in Fig. S3f. At pH 7.4, cumulative DOX release reached only 10% over 400 minutes, whereas at pH 5.3, this value approximately doubled—indicating pH-responsive release behavior. This phenomenon is likely due to acid-induced protonation of DOX. Additionally, the relatively low drug release may stem from strong binding interactions between MGO-HA-FA and DOX, which hinder drug diffusion from the nanoparticles into the release medium. Notably, none of the release profiles exhibited a burst release effect.

Furthermore, we assessed how laser irradiation affects drug release when introduced into the system. As shown in Fig. 3c, the cumulative DOX release of DOX/MGO-HA-FA at pH 5.3 with NIR irradiation reached 22.67% over 400 min, respectively, higher than without NIR irradiation (17.26%). A quicker release can be observed at NIR irradiation, owing to the high optical and thermal conversion efficiency of MGO-HA-FA nanoparticles can accelerate the release of DOX by laser radiation. These results exhibited that DOX/MGO-HA-FA drug release can be considered as having pH/NIR double-release characteristics.

### 3.5. Hemolysis test

**Fig. 4:**
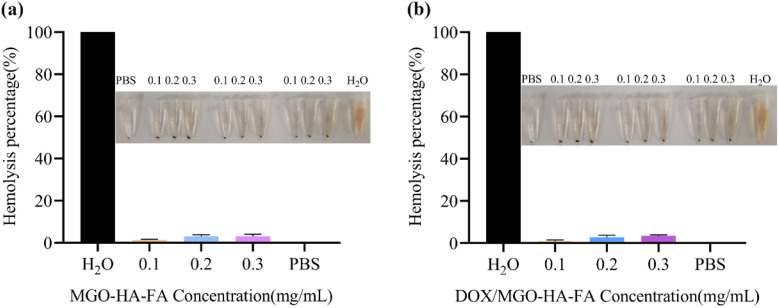
The hemolysis assays of MGO-HA-FA (a) and DOX/MGO-HA-FA (b). Digital photographs of erythrocyte hemolysis with different concentrations of MGO-HA-FA and DOX/MGO-HA-FA.

Fig. 4 presents the hemolysis percentages and corresponding visual hemolysis images of MGO-HA-FA (Fig. 4a) and DOX/MGO-HA-FA (Fig. 4b) at concentrations ranging from 0.1 to 0.3 mg mL^−1^, with deionized water (100% hemolysis, positive control) and PBS (0% hemolysis, negative control) as references. In the MGO-HA-FA group (Fig. 4a), the hemolysis percentages at 0.1, 0.2, and 0.3 mg mL^−1^ were all <2% (far below the 5% threshold for biocompatible materials^50^). In the DOX/MGO-HA-FA group (Fig. 4b), the hemolysis percentages of the drug-loaded nanocomposites remained <2% across all tested concentrations. Consistently, the visual images (insets of Fig. 4a and b) show that the supernatants of MGO-HA-FA/DOX/MGO-HA-FA groups were as pale and transparent as the PBS control (no red hemoglobin release), while the deionized water control exhibited significant red discoloration (complete hemolysis). These results demonstrate that both the blank nanocarrier (MGO-HA-FA) and the drug-loaded system (DOX/MGO-HA-FA) have excellent blood compatibility. The low hemolysis rate (<2%) confirms that the nanocomposites do not damage red blood cell membranes even at the highest tested concentration (0.3 mg mL^−1^), which provides a critical safety guarantee for their intravenous administration in subsequent *in vivo* therapeutic applications.

### 3.6. Cellular uptake of DOX/MGO-HA-FA

**Fig. 5:**
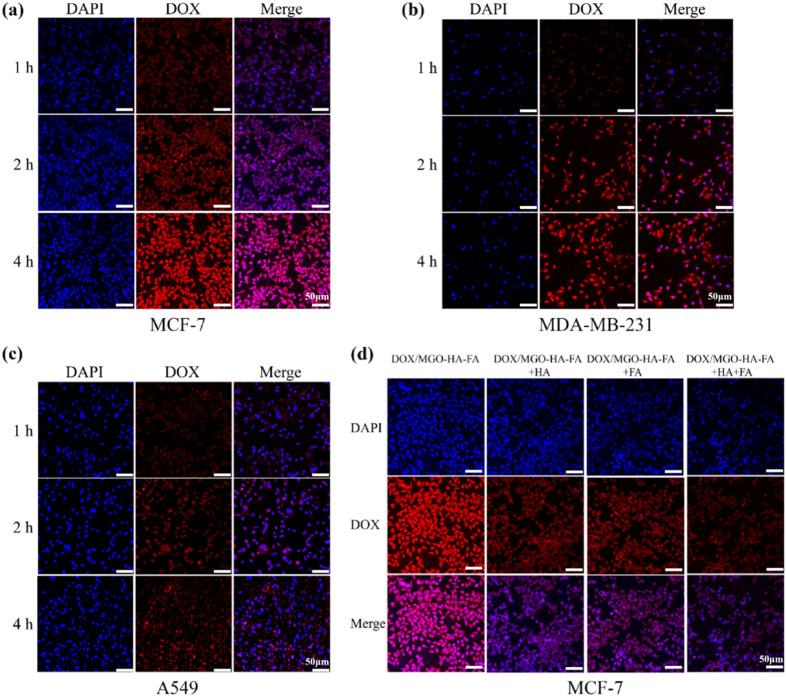
CLSM images of MCF-7 cells (a), MDA-MB-231 cells (b) and A549 cells (c) with DOX/MGO-HA-FA (10 µg mL^−1^) for 1 h, 2 h and 4 h; CLSM images of MCF-7 cells with DOX/MGO-HA-FA, DOX/MGO-HA-FA + HA, DOX/MGO-HA-FA + FA and DOX/MGO-HA-FA + HA + FA. The nuclei stained with DAPI were blue, and the red fluorescence resulted from free DOX. Scale bar: 50 µm.

The targeting capacity of DOX/MGO-HA-FA nanoparticles was assessed *via in vitro* cellular uptake assays. Taking advantage of DOX's autofluorescence, laser confocal microscopy was employed to characterize the cellular uptake of DOX/MGO-HA-FA. As depicted in Fig. 5, red fluorescence intensity increased progressively with extended incubation (up to 4 h), indicating greater internalization of DOX/MGO-HA-FA nanoparticles over time. Notably, uptake of DOX/MGO-HA-FA by MCF-7 cells (Fig. 5a) and MDA-MB-231 cells (Fig. 5b) was significantly higher than by A549 cells (Fig. 5c), given that A549 cells are folate receptor (FR)-negative. Additionally, MCF-7 cells exposed to DOX/MGO-HA-FA showed prominent red fluorescence. In contrast, DOX/MGO-HA-FA + HA + FA group showed the weakest red fluorescence compared to DOX/MGO-HA-FA, DOX/MGO-HA-FA + HA, and DOX/MGO-HA-FA + FA groups (Fig. 5d), suggesting that pretreatment with HA and FA competitively bound to CD44 receptors and FRs. Taken together, these results confirm that DOX released from DOX/MGO-HA-FA is internalized by cells, and functionalizing with HA and FA significantly boosts the targeting potential of DOX/MGO-HA-FA.

### 3.7. CPT synergetic therapy of DOX/MGO-HA-FA *in vitro*

**Fig. 6:**
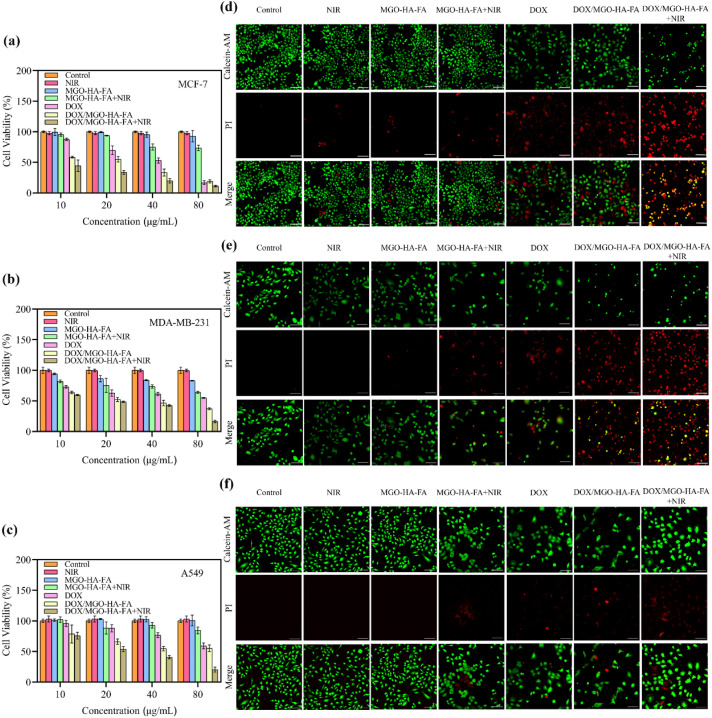
Viability of MCF-7 cells (a), MDA-MB-231 cells (b) and A549 cells (c) with different concentrations of free DOX, MGO-HA-FA and DOX/MGO-HA-FA without laser irradiation and with 808 nm laser (2W cm^−2^) irradiation; live/dead confocal images of MCF-7 cells (d), MDA-MB-231 cells (e) and A549 cells (f) after different treatments *via* the Calcein-AM/PI double staining kits staining at the concentration of 10 µg mL^−1^, Bar = 100 µm.

The CCK-8 assay was conducted to assess the cytotoxicity of DOX/MGO-HA-FA, aiming to verify whether the nanosystem exhibits photothermal/chemotherapy synergy. Cytotoxic effects of MGO-HA-FA, free DOX, and DOX/MGO-HA-FA were investigated at concentrations of 10, 20, 40, and 80 µg mL^−1^ (with or without laser irradiation) on MCF-7, MDA-MB-231, and A549 cells. As shown in Fig. 6a–c, neither standalone NIR irradiation nor incubation with MGO-HA-FA (even at 80 µg mL^−1^) exhibited significant cytotoxicity, confirming excellent biocompatibility. Notably, MGO-HA-FA cytotoxicity increased significantly in a dose-dependent manner following 5-minute irradiation at 808 nm. Compared to DOX/MGO-HA-FA group without light, the cancer-inhibiting capacity of the light-exposed group was enhanced. At a DOX/MGO-HA-FA concentration of 80 µg mL^−1^, only ∼11% of MCF-7 cells and ∼16% of MDA-MB-231 cells remained viable in the photothermal chemotherapy (CPT) group, demonstrating that the strong photothermal effect of DOX/MGO-HA-FA can inhibit *in vitro* tumor cell growth. In contrast, A549 cell viability was 20%, reflecting the targeting capacity of MGO-HA-FA.

Furthermore, the synergistic CPT treatment with DOX/MGO-HA-FA was visually assessed *via* live/dead staining assays. As observed in Fig. 6d–f, minimal red fluorescence was detected in both the standalone NIR group and MGO-HA-FA group, confirming that pure NIR irradiation is safe and MGO-HA-FA possesses excellent biocompatibility. Notable red fluorescence was observed in MGO-HA-FA + NIR, free DOX, and DOX/MGO-HA-FA groups, indicating that standalone photothermal treatment or chemotherapy induces some cancer cell death. Among these, CPT combination therapy exhibited the strongest inhibitory effect, consistent with earlier findings. These results confirm that DOX/MGO-HA-FA is a promising nanosystem for synergistic PTT/chemotherapy.

### 3.8. Antitumor efficacy assay of DOX/MGO-HA-FA

**Fig. 7:**
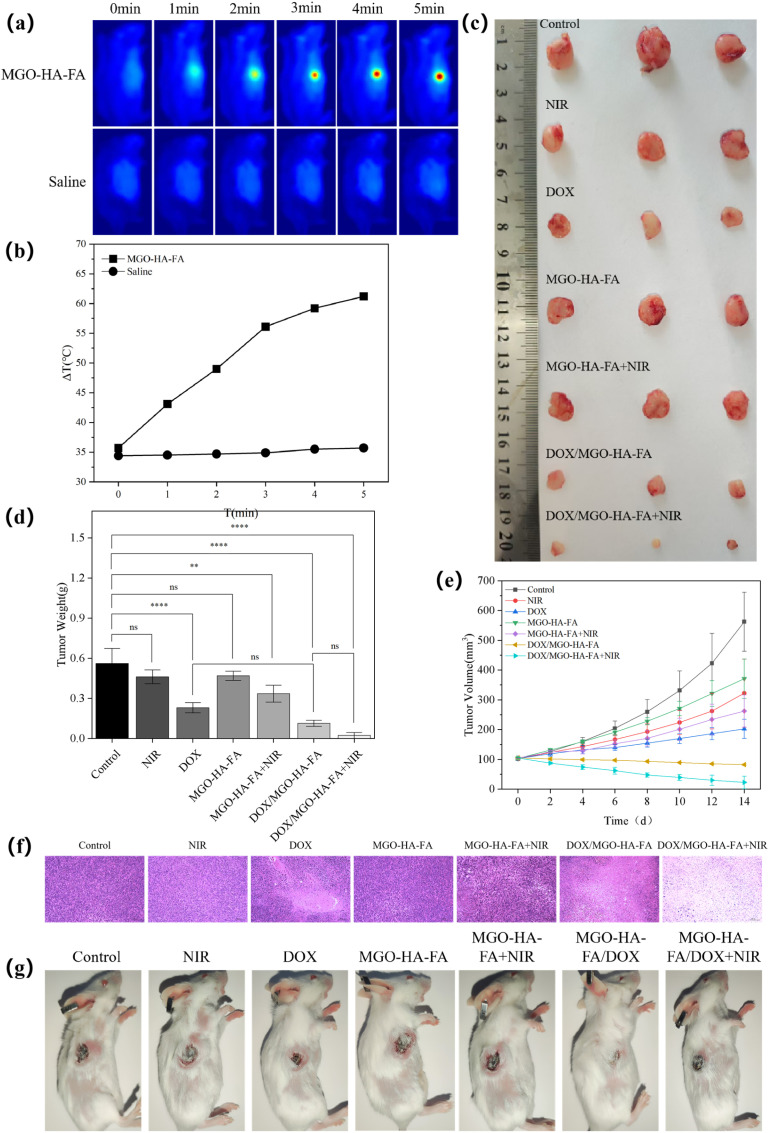
*In vivo* antitumor efficacy of DOX/MGO-HA-FA. Thermal imaging of tumor sites treated with PBS and MGO-HA-FA under NIR irradiation (808 nm, 2 W cm^−2^) for 5 min (a); the quantitative analysis of temperature variation of tumors based on (b); representative images of excised tumors from different treatment groups (c); quantitative analysis of tumor weight post-treatment (d); tumor volume progression curves (e); images of H&E staining of tumor slices. Scale bar = 100 µm(f); tumor photographs after 14 days of different treatment (g).

A subcutaneous 4T1 breast tumor model was established in BALB/c mice to assess the antitumor activity of DOX/MGO-HA-FA. Treatments were initiated once tumor volumes reached ∼100 mm^3^. Mice were randomly divided into seven groups (*n* = 3 per group): (a) control (normal saline); (b) NIR laser alone; (c) free DOX; (d) MGO-HA-FA carrier alone; (e) MGO-HA-FA + NIR; (f) DOX/MGO-HA-FA alone; (g) DOX/MGO-HA-FA + NIR. All formulations were administered *via* tail vein injection at a dose of 5 mg kg^−1^. As shown in Fig. 7a and b, tumor site temperatures in saline-treated mice remained stable at 35.7 °C under 808 nm NIR laser irradiation. In contrast, MGO-HA-FA-treated mice exhibited a time-dependent temperature rise, reaching 56.1 °C at 3 min and peaking at 61.2 °C within 5 min. These findings confirm the strong *in vivo* photothermal conversion efficiency of MGO-HA-FA, as the temperature elevation to over 50 °C is sufficient to induce irreversible damage to tumor cells while sparing normal tissues due to the localized photothermal effect.

Further examination of tumor weight in Fig. 7c and d revealed no significant differences among saline, saline + NIR, and MGO-HA-FA groups, indicating that neither MGO-HA-FA nor NIR irradiation individually exhibited significant inhibitory effects on tumor growth. The average tumor weight in MGO-HA-FA + NIR and free DOX groups was 0.33 g, indicating limited inhibitory efficacy of monotherapy regimens—likely due to the insufficient drug accumulation of free DOX in tumor sites and the single-mode photothermal damage induced by MGO-HA-FA + NIR. In contrast, DOX/MGO-HA-FA reduced tumor weight to 0.11 g (50% smaller than free DOX), indicating enhanced tumor targeting efficacy of the nanocomposite *via* the dual-receptor (CD44 and folate receptor) mediated endocytosis, which facilitates selective drug delivery to tumor cells and reduces systemic side effects. Most notably, DOX/MGO-HA-FA + NIR achieved the smallest weight (0.02 g), highlighting the synergistic effect of chemophotothermal therapy: the NIR-triggered local hyperthermia not only directly ablates tumor cells but also enhances the permeability of tumor cell membranes, accelerating the intracellular release and diffusion of DOX to amplify the chemotherapeutic efficacy.

Dynamic tumor volume monitoring over 14 days (Fig. 7e) showed the control group had the largest average volume (562 mm^3^), whereas MGO-HA-FA + NIR and free DOX groups exhibited volumes of 262 mm^3^ and 202 mm^3^, respectively, confirming the modest tumor suppression of monotherapies. DOX/MGO-HA-FA further reduced the volume to 82 mm^3^, and DOX/MGO-HA-FA + NIR achieved the minimal volume (22 mm^3^), demonstrating its enhanced therapeutic effectiveness; this sustained tumor growth inhibition can be attributed to the prolonged circulation time of the nanocarrier in the bloodstream and the synergistic action of chemotherapy and photothermal therapy.

Histopathological analysis *via* H&E staining (Fig. 7f) revealed no necrosis in control and MGO-HA-FA groups, whereas the NIR, free DOX, and MGO-HA-FA + NIR groups exhibited merely localized damage confined to small necrotic areas, consistent with their moderate tumor inhibition outcomes. Notably, DOX/MGO-HA-FA + NIR elicited significant cellular alterations, characterized by nuclear shrinkage, fragmentation, and diminished cell density, concomitant with extensive necrotic areas and cavitation, thereby demonstrating marked cytotoxicity against breast tumor cells. The trend observed in the external photographs of mouse tumors is consistent with the findings from H&E staining (Fig. 7g), where tumors in the DOX/MGO-HA-FA + NIR group showed the most significant regression with no obvious recurrence, whereas tumors in other groups remained larger in size or showed partial regrowth.

Collectively, these findings demonstrate that DOX/MGO-HA-FA in combination with NIR laser irradiation enables synergistic chemophotothermal therapy, exhibiting superior efficacy in inhibiting tumor growth compared to monotherapies and highlighting significant therapeutic benefits. This dual-modal therapeutic strategy leverages the targeted drug delivery capability of the HA-FA functionalized nanocarrier and the localized photothermal effect, providing a promising platform for the efficient treatment of breast cancer with minimized systemic toxicity.

### 3.9. The biosafety of DOX/MGO-HA-FA

**Fig. 8:**
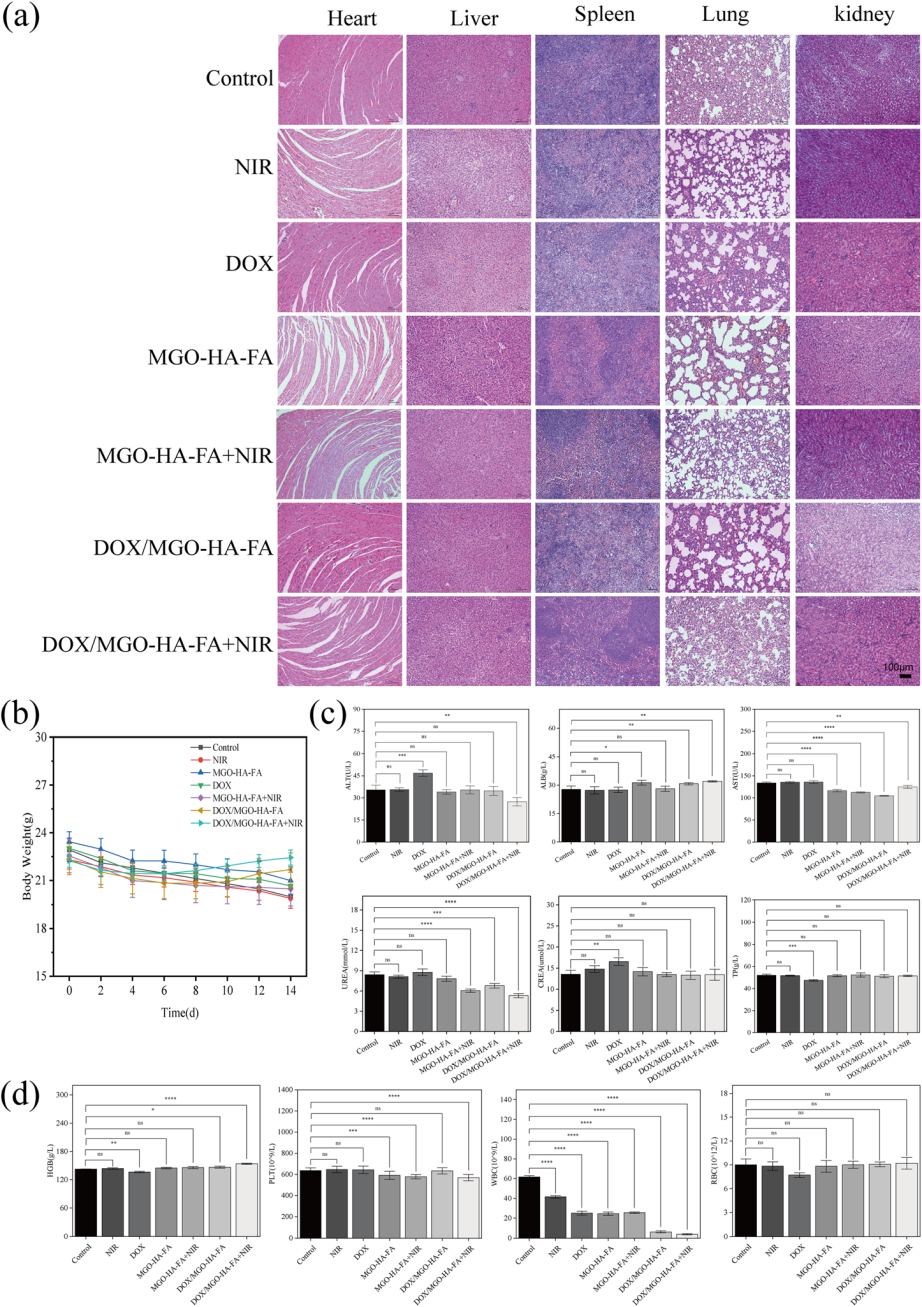
The safety and biocompatibility of DOX/MGO-HA-FA *in vivo*. Representative images of H&E staining of the major organs (a); body weight dynamics of mice during the treatment period (b); the pivotal blood biochemical indicators of BALB/c mice (c); the pivotal blood routine indicators of BALB/c mice (*n* = 3 mice/group) after 14 days of different treatment. Data are presented as mean ± SD (ns > 0.05, *p < 0.05, **p < 0.01) (d).

The biosafety of DOX/MGO-HA-FA in the therapeutic setting was further validated, with three core safety endpoints summarized in Fig. 8: histopathology of major organs, body weight dynamics, and hematological/biochemical profiles. First, hematoxylin and eosin (H&E) staining of heart, liver, spleen, lung, and kidney tissues (Fig. 8a) revealed intact architecture with no significant pathological damage across all groups, confirming that DOX/MGO-HA-FA did not induce off-target organ injury. Second, serial body-weight monitoring (Fig. 8b) showed no statistically significant alterations among all treatment groups; notably, mice in the DOX/MGO-HA-FA and DOX/MGO-HA-FA + NIR groups exhibited gradual weight gain in the late treatment stage, verifying favorable treatment tolerability. Third, blood biochemical assays (liver function: ALT, AST, ALB, TP; renal function: CREA, UREA; Fig. 8c) and complete blood count (CBC) analysis (HGB, PLT, RBC, WBC; Fig. 8d) demonstrated no significant differences between DOX/MGO-HA-FA treatment groups and the control group. In contrast, the free DOX group presented moderate elevation of ALT/AST, consistent with its inherent hepatorenal toxicity, while NIR laser irradiation alone exerted negligible effects on all parameters. The decreased WBC counts in DOX/MGO-HA-FA groups were likely attributed to alleviated tumor-induced inflammation from effective tumor suppression. Collectively, these results confirm that DOX/MGO-HA-FA mediates targeted tumor drug delivery, minimizes systemic drug exposure and off-target toxicity, and achieves optimal *in vivo* therapeutic efficacy with a favorable safety profile.

## 4. Conclusions

**Scheme 1:**
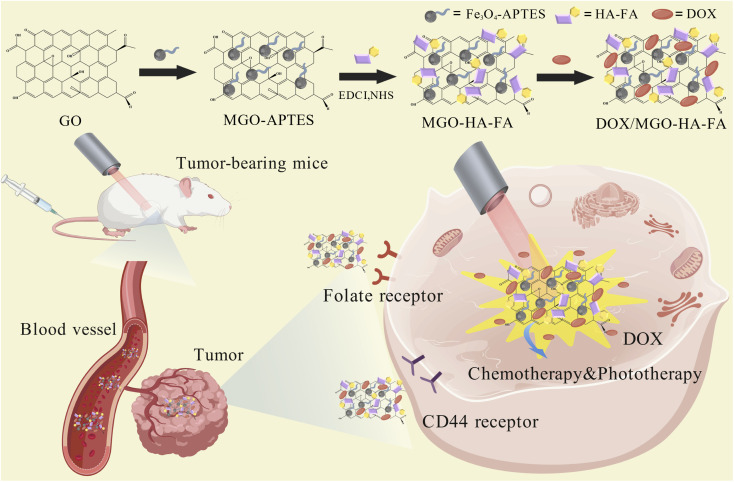
Schematic illustration of the synthesis and therapeutic mechanism of DOX/MGO-HA-FA.

In conclusion, we successfully designed and fabricated MGO-HA-FA nanocomposites, which serve as an efficient nanoplatform integrating multi-targeting capabilities with chemophotothermal therapy for cancer treatment. DOX/MGO-HA-FA system was constructed by conjugating HA-FA polymer-coated Fe_3_O_4_-graphene oxide and achieving efficient DOX loading. Upon internalization into cancer cells, DOX/MGO-HA-FA undergoes rapid temperature elevation following 5-minute NIR irradiation, and cell proliferation is significantly suppressed *via* synergistic chemophotothermal therapy (Scheme 1). This combined photochemical therapy exerted a potent inhibitory effect on tumor cells (with a tumor inhibition rate of approximately 90%), outperforming the therapeutic efficacy of standalone photothermal therapy or chemotherapy.

*In vivo* studies employing a breast cancer model confirmed the marked antitumor efficacy of DOX/MGO-HA-FA, demonstrating significant suppression of tumor progression while maintaining physiological parameters within normal ranges for body weight, hematological indices, and major organ functions. These results substantiate the nanoplatform's favorable safety profile and underscore its therapeutic potential. In summary, this study offers a viable multi-targeted nanoplatform with combined chemo-photothermal capabilities for efficient cancer therapy.

## Author contributions

Bin Jia: validation, writing – review & editing, conceptualization. Yimu Zhong: writing – original draft, methodology, investigation, data curation. Danyang Zhai: methodology, investigation. Jing Pang: writing – original draft, methodology, investigation, data curation. Bo Sha: investigation, data curation. Na Li: investigation, data curation. Bo Li: writing – review & editing. Wenting Liang: writing – review & editing, validation. Wei Bian: writing – review & editing.

## Conflicts of interest

The authors declare no conflict of interest.

## Supplementary Material

RA-016-D5RA08760K-s001

## Data Availability

The authors confirm that the data supporting the findings of this study are available within the article and its supplementary information (SI). Supplementary information: materials and experimental methods (synthesis of MGO-HA-FA and DOX/MGO-HA-FA), kinetic and isotherm adsorption models (eqn (S1)–(S4)), photothermal conversion efficiency calculation (formula (S1)), drug loading and release calculations (formulas (S2) and (S3)), Tables S1 and S2 (kinetic and isotherm parameters), Fig. S1–S3 (synthetic route, FTIR spectra, and drug loading/release profiles), and detailed cell culture and uptake procedures. See DOI: https://doi.org/10.1039/d5ra08760k.
