# Supplementary material for: Fe3O4-graphene oxide nanocomposites functionalized with hyaluronic acid and folic acid as dual pH/NIR-responsive platforms for synergistic chemophotothermal therapy of breast cancer
Source: RSC Adv. 2026 Mar 2;16(13):11580–93. doi: 10.1039/d5ra08760k (PMC12951213; doi:10.1039/d5ra08760k)
Supplement: RA-016-D5RA08760K-s001 [file RA-016-D5RA08760K-s001.pdf]

Supplementary Information (SI) for RSC Advances.
This journal is © The Royal Society of Chemistry 2026

Supplementary Material

Fe_3O_4-Graphene Oxide Nanocomposites Functionalized with Hyaluronic Acid and Folic Acid as Dual pH/NIR-Responsive Platforms for Synergistic Chemophotothermal Therapy of Breast Cancer

Bin Jia [a,*], Yimu Zhong [a,b], Jing Pang [a,c], Bo Sha [a], Danyang Zhai [a], Na Li [a], Bo Li [a], Tao Gong [c,*], Wei Bian [a,*]

[a] Department of Chemistry, School of Basic Medical Science, Shanxi Medical University, Taiyuan, 030001, China

[b] Academy of Medical Sciences, Shanxi Medical University, Taiyuan, 030001, China

[c] Department of Biochemistry and Molecular Biology, School of Basic Medical Science, Shanxi Medical University, Taiyuan, 030001, China

[*] Corresponding authors. E-mail addresses: bjia2006@163.com (Bin Jia), gyt830626@163.com (Tao Gong), weibian@sxmu.edu.cn (Wei Bian).

1.Materials

Folic acid (FA), Hyaluronic acid (HA)(The molecular weight of hyaluronic acid (HA) used in this study is 50 kDa), graphene oxide (GO), dicyclohexyl carbodiimide (DCC), n-hydroxysuccinylimide (NHS), 3-aminopropyl triethoxysilane (APTES), carbodiimide hydrochloride (EDCI) and formamide were all purchased from Macklin (Shanghai, China). Dimethyl sulfoxide (DMSO), ammonia solution (NH_3, 25%), and anhydrous ethanol were supplied by Tianjin Reagent Factory. Doxorubicin hydrochloride (DOX) was purchased from Aladdin Industrial Corporation. Unless otherwise specified, all other chemicals were of analytical reagent grade.

2.Preparation of MGO-HA-FA and DOX/MGO-HA-FA

2.1. The synthesis of the MGO-HA-FA nanoparticles

The synthesis of aminated MGO (MGO-APTES) was accomplished based on the previously published literature. HA-FA polymer was synthesized by the reaction of the carboxylic acid with alcohol (Scheme 1) [1-3], according to a previously reported method [4]. The synthetic strategy of MGO-HA-FA is as follows. First, HA-FA (100 mg) was dissolved in DI water/DMF (20 mL) in the ratio of 1:1, then EDCI-NHS (0.5 mmol) was added into the above solution with stirring for 3 h to re-activate the carboxyl groups of supramolecular polymers. Thereafter, the prepared amino-MGO (100 mg) was added to the above aforesaid suspension, which was mechanically stirred for 36 h at room temperature. The obtained product was separated with a permanent magnet repeatedly and rinsed with anhydrous ethanol to remove the unreacted reactants. Finally, The synthetic route of the HA-FA polymer is illustrated

in Figure S1, the product (MGO-HA-FA) was acquired by freeze-drying.The conjugation of HA and FA was achieved via amide bond formation, not ester bond. Briefly, the carboxyl groups of FA were activated using 1-ethyl-3-(3-dimethylaminopropyl) carbodiimide (EDC) and N-hydroxysuccinimide (NHS) under mild conditions (pH 5.5). Meanwhile, the primary hydroxyl groups of HA were not activated; instead, the reaction was specifically directed to the carboxyl group of FA and the amino groups introduced on HA via modification with ethylenediamine. This strategy avoided unwanted side reactions between the carboxyl groups of HA and FA, ensuring efficient conjugation.

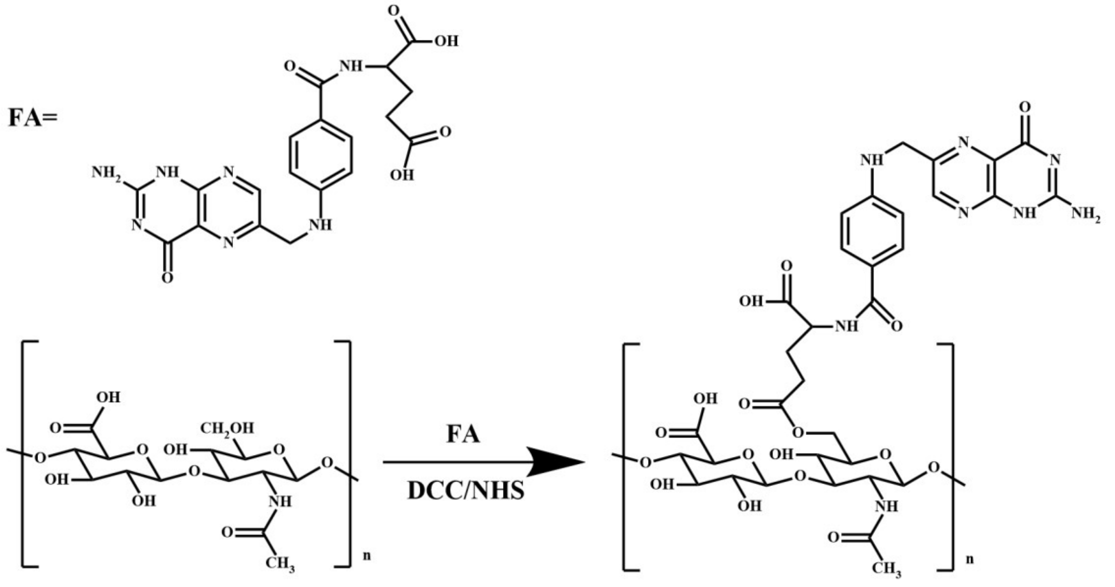

HA HA-FA

Figure S1. Synthetic Route of the HA-FA Polymer

2.2. The synthesis of the DOX/MGO-HA-FA nanoparticles

For drug loading, MGO-HA-FA (10 mg) was dispersed in a phosphate buffer solution (PBS, pH 7.4) and ultrasonically treated to form a homogeneous dispersion. DOX/MGO-HA-FA were prepared by adding 5 mL (0.1 mg mL^{-1}) of DOX solution

to the dispersion, then incubated for 24 h with continuous stirring at 37℃ in the dark, the unloaded solution was removed by magnetic separation and the products were washed with PBS solution three times and finally freeze dried.

3.Lagergren's pseudo-first-order kinetic model (Equation S1) and Ho's pseudo-second-order kinetic model (Equation S2).

$$\ln(q_e - q_t) = \ln q_e - k_1 t \tag{1}$$

$$\frac{t}{q_t} = \frac{1}{k_2 q_e^{2}} + \frac{t}{q_e} \tag{2}$$

where, in Equation (1) and (2): q_e (mg g^{-1}) is equilibrium adsorption efficiency; q_t (mg g^{-1}) is the drug loading efficiency at different time points; t (min) is the drug loading time; k_1 and k_2 are kinetic constants.

4. Langmuir isotherm adsorption model (Equation S3) and Freundlich isotherm adsorption model (Equation S4).

$$\frac{C_e}{q_e} = \frac{C_e}{q_m} + \frac{1}{q_m K_L} \tag{3}$$

$$\ln q_e = \ln K_f + \frac{1}{n} C_e \tag{4}$$

where, in Equation (3) and (4): C_e (mg L^{-1}) is the mass concentration at balanced drug loading; q_m (mg g^{-1}) is the drug loading efficiency at saturation; q_e (mg g^{-1}) is the drug loading efficiency at equilibrium; K_L (L mg^{-1}) is the dissociation constant; K_f (L g^{-1}) is the Freundlich constant; n^{-1} is the Freundlich component factor.

Formula S1. Photothermal conversion efficiency calculation

$$\eta = \frac{hS(T_{max}-T_{surr})-Q_{dis}}{I(1-10^{-A_{\lambda}})} \times 100\%$$

where hS denotes the heat dissipation coefficient of the system, T_{max} is the maximum temperature of the solution under laser irradiation, T_{surr} represents the ambient temperature, Q_{dis} is the background thermal contribution of the solvent, I denotes the incident laser power density, and A_{λ} represents the absorbance of the material at the laser wavelength λ.

Formula S2. Drug loading capacity calculation.

$$\text{DLC (wt\%)} = \frac{W_{loaded}}{W_{loaded} + W_{material}} \times 100\% \quad (1)$$

where, W_{loaded} and $W_{material}$ represent the weight of loaded DOX and weight of MGO-HA-FA nanoparticles, respectively.

Formula S3. Drug release percentage calculation.

$$\text{The percentage of drug released} = \frac{m_{\text{the released amounts of drug}}}{m_{\text{the loaded amounts of drug}}} \times 100\% \quad (2)$$

where, $m_{\text{the released amounts of drug}}$ and $m_{\text{the loaded amounts of drug}}$ represent the amount of released DOX and the amount of total loaded DOX, respectively.

Table S1. The kinetic parameters for DOX loaded by MGO-HA-FA.

Lagergren's pseudo-first-order model			Ho's pseudo-second-order model		
q_e (mg g^{-1})	k_1 (h^{-1})	R^2	q_e (mg g^{-1})	k_2 (g mg^{-1} min^{-1})	R^2
8.49	0.40007	0.89756	33.17	0.10950	0.99955

Table S2. The adsorption isotherm parameters for DOX loaded by MGO-HA-FA.

Langmuir isotherm model			Freundlich isotherm model		
q_m (mg g^{-1})	K_L (L mg^{-1})	R^2	n	K_f (L g^{-1})	R^2
3846	0.0083	0.6380	10.96	37.13	0.92712

5.Anti-tumor activity of MGO-HA-FA *in vitro*

5.1. Cell lines

The MCF-7 cells, MDA-MB-231 cells, and A549 cells were generously provided by Shanxi Medical University. Human breast cancer cell lines MCF-7 (CD44 receptor +, FA receptor +) were grown in RPMI 1640 medium, supplemented with 10% fetal calf serum (FBS) and 1% penicillin-streptomycin. The MDA-MB-231 (CD44 receptor +, FA receptor +), a triple-negative breast cancer cell line, was cultured in a DMEM-high glucose medium containing 10% FBS and 1% penicillin-streptomycin. Human lung cancer cells A549 (CD44 receptor +, FA receptor -) were maintained in Mccoy's 5A medium supplemented with FBS (10%) and 1% penicillin-streptomycin. All cell lines were incubated in a humidified atmosphere of 5% CO_2 at 37°C. The cells used for the experiments were in their exponential growth phase.

5.2. Intracellular uptake

5.2.1. Time-dependent cell uptake

To study the endocytosis of cancer cells on DOX/MGO-HA-FA nanoparticles, the MCF-7 cells, MDA-MB-231 cells, and A549 cells were seeded into confocal dishes (2×10^5 cells per well) overnight. After removing the culture medium, the cells were incubated with media containing 10 μg/mL DOX/MGO-HA-FA for 1, 2, and 4 h. At determined time points, the medium was removed and washed with the PBS solution to stop the cellular uptake, and the nuclei of the cells were stained with DAPI. Finally, fluorescence images were viewed by a confocal laser scanning microscope. The blue fluorescence indicates the nucleus labeled with DAPI, while the red

fluorescence reflects DOX's intrinsic fluorescence.

5.2.2. Receptor-dependent cell uptake

MCF-7 cells were plated into confocal dishes. After cell adhesion, separately replaced old medium with medium containing 5 μg/mL HA, 5 μg/mL FA, and 5 μg/mL HA+5 μg/mL FA for 1 h, then incubated with DOX/MGO-HA-FA for 4 h. Same as above, observe red fluorescence intensity.

5.3. Anti-cancer activity evaluation of DOX/MGO-HA-FA mediated PTT/chemo-therapy

The CCK8 assays were used to determine the *in vitro* cytotoxicity of MGO-HA-FA and DOX/MGO-HA-FA. Briefly, the well-growing MCF-7 cells, MDA-MB-231 cells, and A549 cells were cultured in 96-well plates at 8×10^3 cells/well for 24 h to make the cells firmly adherent. Afterward, replaced the old medium with 100 μL of fresh culture medium containing different concentrations (10, 20, 40, and 80 μg/mL) of MGO-HA-FA and DOX/MGO-HA-FA nanoparticles as follows groups: (i) Control, (ii) NIR; (iii) MGO-HA-FA; (iv) MGO-HA-FA + NIR; (v) DOX; (vi) DOX/MGO-HA-FA; (vii) DOX/MGO-HA-FA + NIR. Then incubated for 4 h, +NIR (808 nm, 2 W/cm^2) groups were irradiated with 5 min for thermal therapy, and further incubated for 20 h. At the indicated times, the culture medium was removed and the wells were washed three times with PBS. Then CCK8 assay reagents were added to each well and the absorbance of each well was finally measured at 450 nm after 4 h on an enzyme-linked immunoassay instrument.

Subsequently, the double staining with Calcein-AM/PI was applied to visually

observe live and dead cells induced by DOX/MGO-HA-FA. In a nutshell, after inoculating MCF-7 cells, MDA-MB-231 cells, and A549 cells in 24-well plates at a density of 1×10^5 cells per well for 24 h, they were treated following the aforementioned groups. The medium was removed and rinsed at least three times with PBS. Then, the prepared staining solution was added to each well for staining at 37 °C for 15 min in the dark. Lastly, observe the fluorescence of cells through a confocal laser scanning microscope. The living cells showed green fluorescence and red fluorescence for dead cells.

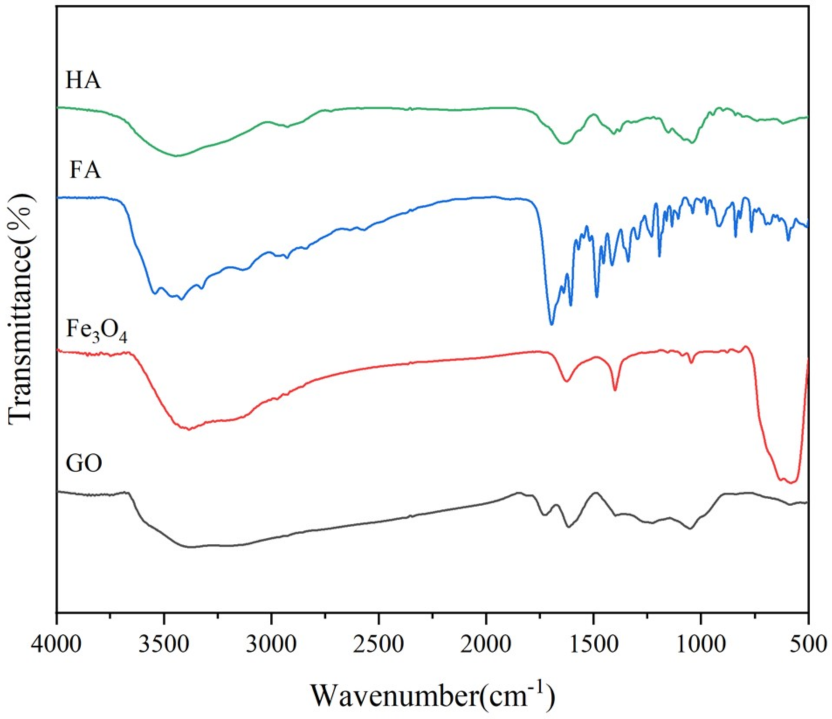

Figure. S2 FTIR spectra of GO, Fe_3O_4, FA and HA

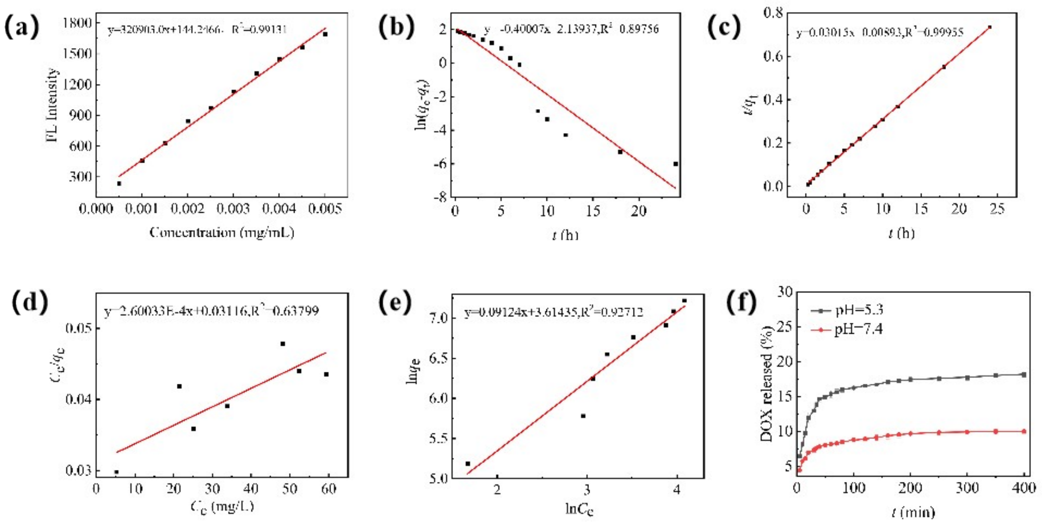

Figure. S3 The standard concentration curve of the DOX hydrochloride (a); The linear fitting curves of the Lagergren's quasi-first-order kinetic model (b) and Ho's quasi-second-order kinetic model (c) for DOX loaded by MGO-HA-FA; The linear fitting curves of single-layer Langmuir model (d) and the multilayer Freundlich adsorption model (e) for DOX loaded by MGO-HA-FA; The cumulative release of DOX on MGO-HA-FA in PBS buffer at pH 7.4 and 5.3 at 37 °C (f).

[1] W. Liang, Y. Huang, D. Lu, X. Ma, T. Gong, X. Cui, B. Yu, C. Yang, C. Dong, S. Shuang, β-Cyclodextrin–Hyaluronic Acid Polymer Functionalized Magnetic Graphene Oxide Nanocomposites for Targeted Photo-Chemotherapy of Tumor Cells, Polymers (Basel), 11 (2019).

[2] S. Jiang, H. Li, L. Zhang, W. Mu, Y. Zhang, T. Chen, J. Wu, H. Tang, S. Zheng, Y. Liu, Y. Wu, X. Luo, Y. Xie, J. Ren, Generic Diagramming Platform (GDP): a comprehensive database of high-quality biomedical graphics, Nucleic Acids Research, 53 (2025) D1670-D1676.

[3] C. Wen, R. Cheng, T. Gong, Y. Huang, D. Li, X. Zhao, B. Yu, D. Su, Z. Song, W. Liang, β-Cyclodextrin-cholic acid-hyaluronic acid polymer coated Fe(3)O(4)-graphene oxide nanohybrids as local chemo-photothermal synergistic agents for enhanced liver tumor therapy, Colloids Surf B Biointerfaces, 199 (2021) 111510.

[4] Y. J. Zhou, W. J. Wan, Y. Tong, M. T. Chen, D. D. Wang, Y. Wang, B. G. You, Y. Liu, X. N. Zhang, Stimuli-responsive nanoparticles for the codelivery of chemotherapeutic agents doxorubicin and siPD-L1 to enhance the antitumor effect, J Biomed Mater Res B Appl Biomater, 108 (2020) 1710-1724.
